# Dietary calcium intake and mortality risk from cardiovascular disease and all causes: a meta-analysis of prospective cohort studies

**DOI:** 10.1186/s12916-014-0158-6

**Published:** 2014-09-25

**Authors:** Xia Wang, Hongxia Chen, Yingying Ouyang, Jun Liu, Gang Zhao, Wei Bao, Maosheng Yan

**Affiliations:** Department of Maternal and Child Health Care, School of Public Health, Shandong University, Jinan, China; Department of Nutrition and Food Hygiene, School of Public Health, Tongji Medical College, Huazhong University of Science and Technology, 13 Hangkong Road, Wuhan, 430030 China; Institute of Biomedicine, Taihe Hospital, Hubei University of Medicine, Shiyan, Hubei Province China; Department of Cardiovascular Sciences, Shandong Provincial Hospital affiliated to Shandong University, Jinan, China; Guangdong Provincial Key Laboratory of Occupational Disease Prevention and Treatment, Guangdong Provincial Hospital for Occupational Disease Prevention and Treatment, 68 Haikang Road, Guangzhou, 510300 China

**Keywords:** Calcium, Cardiovascular disease, Cohort, Meta-analysis, Mortality

## Abstract

**Background:**

Considerable controversy exists regarding the association between dietary calcium intake and risk of mortality from cardiovascular disease and all causes. Therefore, we performed a meta-analysis of prospective cohort studies to examine the controversy.

**Methods:**

We identified relevant studies by searching MEDLINE, Embase, and the Cochrane Library databases between 1 September 2013 and 30 December 2013. Reference lists of relevant articles were also reviewed. Observational prospective studies that reported relative risks and 95% confidence intervals for the association of calcium intake with cardiovascular and all-cause mortality were eligible. Study-specific relative risks were pooled using a random-effects model.

**Results:**

In this meta-analysis, 11 prospective studies with 12 independent cohorts, involving 757,304 participants, were eligible. There was evidence of a non-linear association between dietary calcium intake and risk of mortality from cardiovascular disease (*P* for non-linearity <0.01) and all causes (*P* for non-linearity <0.01). A dose-response analysis showed a U-shaped relationship between dietary calcium intake and cardiovascular mortality. Intakes that were lower and higher than around 800 mg/day were gradually associated with a higher risk of cardiovascular mortality. For all-cause mortality, we also observed a threshold effect at intakes around 900 mg/day. The risk of all-cause mortality did not decrease further at intakes above 900 mg/day.

**Conclusions:**

This meta-analysis of prospective cohort studies suggests that dietary calcium intake is associated with cardiovascular mortality in a U-shaped manner and that high dietary calcium intake (>900 mg/day) is not associated with a decreased risk of all-cause mortality.

**Electronic supplementary material:**

The online version of this article (doi:10.1186/s12916-014-0158-6) contains supplementary material, which is available to authorized users.

## Background

Calcium is one of the most common and abundant minerals in the body and has many critical biologic functions. The body tightly controls circulating levels of calcium, usually maintaining a constant range of 1.0 to 1.2 mmol/L [1]. Increasing calcium intake has been recommended by many healthcare professionals because of its proposed benefit for bone health. Consequently, more than 50% of older men and almost 70% of older women in the US are regular users of calcium supplements [2,3].

The health effects of calcium intake on non-skeletal outcomes, including cardiovascular and all-cause mortality, have received growing attention. Some prospective studies found an inverse association between dietary calcium intake and mortality from ischemic heart disease or cardiovascular disease (CVD) [4,5]. By contrast, several recent re-analyses of randomized trials showed that calcium supplements are associated with a higher risk of both ischemic heart disease and stroke, which are two major causes of cardiovascular mortality [6-8]. Thus, the current evidence for an association between calcium intake and risk of cardiovascular and all-cause mortality remains insufficient and controversial. Differences in calcium intake doses, which were higher in the trials, may have led to the differences between the observational studies and randomized trials.

Therefore, to evaluate whether the association between calcium intake and risk of mortality from CVD and all causes varies by levels of calcium intake, we conducted a dose-response meta-analysis of prospective cohort studies.

## Methods

### Search strategy

We followed the Meta-analysis of Observational Studies in Epidemiology [9] for conducting and reporting the present study (Additional file 1). We carried out a meta-analysis of prospective cohort studies that evaluated the association between dietary calcium intake and risk of mortality from CVD and all causes.

We systematically searched databases, including MEDLINE (from 1950), Embase (from 1980), and the Cochrane Library (from 1960), between 1 September 2013 and 30 December 2013 (last date searched). The computer-based searches included the key words ‘calcium intake’, ‘calcium supplements’, ‘calcium supplementation’, ‘cardiovascular mortality’, ‘all-cause mortality’, ‘cause of death’, ‘cardiovascular diseases’, ‘coronary heart disease’, ‘ischemic heart disease’, ‘coronary artery disease’, ‘myocardial infarction’, ‘stroke’, ‘prospective studies’, and ‘follow-up studies’. No language restrictions were imposed on publications. Furthermore, we identified additional articles by manually searching the reference lists of pertinent articles and recent reviews.

### Study selection

To be included, studies had to be prospective cohort studies that reported relative risks (RRs) with 95% confidence intervals (CIs) for the association between dietary calcium intake and mortality from CVD and all causes. We excluded studies with ecological, case-control, or cross-sectional designs; studies with no adjustment for potential confounders; and studies that did not report RRs or hazard ratios and corresponding 95% CIs.

### Data extraction and quality assessment

Data extraction was conducted using a standardized data collection form. The primary exposure variable was dietary calcium intake, which was estimated from foods only, but we also examined supplemental calcium. Outcomes of interest in this study were mortality from CVD and mortality from all causes. All outcomes were classified based on the World Health Organization’s International Classification of Disease criteria. Two authors (JL and YO) independently selected studies and performed the data extraction. We recorded the following characteristics for each identified paper: first author, publication year, cohort name, geographical location, sample size of the cohort and number of outcomes, follow-up (years), age at entry, sex, assessment method of dietary calcium intake, ascertainment of outcomes, variables that entered into the multivariable model as potential confounders, and RRs and the associated measure of variance for all categories of dietary calcium intake. Study quality was evaluated by using the Newcastle-Ottawa quality assessment scale [10]. The system allowed a total score of 0 to 9 points (9 represented the highest quality).

To perform a dose-response meta-analysis, we assigned the median level of calcium intake in each category to the corresponding RR of that category for each study. If medians were not reported, we used the mean level of calcium intake in each category. If the highest category of the studies was open-ended, the difference from the lowest range to the median was considered equivalent to the same difference in the closest adjacent category.

### Data synthesis and analysis

We used the results of the original studies from multivariable models with the most complete adjustment for potential confounders. We utilized a random-effects model to account for inter-study variation and to provide a more conservative effect than a fixed-effects model. Between-study heterogeneity was assessed using the Cochran’s Q test (significance level at *P* <0.10) [11]. *I*^*2*^ was also evaluated to quantify the proportion of inconsistency across studies [12].

We tested for potential non-linearity in the association between calcium intake and mortality from CVD and all causes using a random-effects dose-response meta-analysis [13]. A potential curvilinear relationship was assessed using restricted cubic splines with three knots at fixed percentiles (10%, 50%, and 90%) of the distribution [14]. We first estimated a restricted cubic spline model with a generalized least-squares regression taking into account the correlation within each set of published RRs [13-15]. We then pooled the study-specific estimates using the restricted maximum likelihood method [16]. A *P*-value for a non-linear relationship was calculated by testing the null hypothesis that the coefficient of the second spline was equal to zero [17].

We used STATA version 12.0 (StataCorp LP, College Station, TX, USA) to analyze the data. Except where otherwise specified, *P* <0.05 was considered statistically significant.

## Results

### Literature search

**Figure 1:**
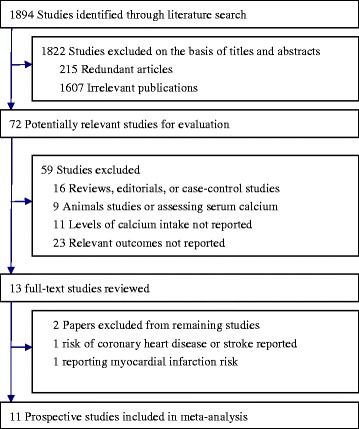
**Flow chart of study selection.** Shows literature search for prospective cohort studies of calcium intake in relation to cardiovascular and all-cause mortality.

Overall, the search initially identified 1,894 reports (Figure 1). After excluding duplicates and papers that did not meet the inclusion criteria, we obtained 13 full articles of potentially relevant studies. After full-text reviews, 2 out of the 13 articles were excluded because they reported risk of coronary heart disease or stroke rather than mortality [18,19]. Finally, 11 articles [5,18,20-28] with 12 independent cohorts fulfilled our inclusion criteria. Dai *et al*.’s report [21] included data from two independent cohorts.

### Study characteristics

Table 1 illustrates the characteristics of the 11 included studies, all of which had prospective cohort design. Of these, nine prospective studies [5,18,20-26] reported data on the relation of dietary calcium intake and total CVD mortality, and six studies [5,20-22,26,28] on all-cause mortality. Six prospective studies [20,22-25,27] examined the relationship between supplemental calcium intake and cardiovascular mortality. One study consisted entirely of men, two of women only, and eight of both men and women. Study length ranged from 5.5 years to 28 years. Three studies were conducted in the US, one in Canada, three in China, and four in Europe.

**Table 1:** **Summary of prospective studies that examined the association between calcium intake and risk of mortality**

**Study**	**Country**	**Number of participants**	**Age (years)**	**Endpoints (Number of cases)**	**Follow-up period and person time** ^**a**^	**Calcium intake (mg/day)**	**Covariates in fully adjusted model**	**Quality score**
Chan *et al*. 2013, a cohort of older community-dwelling people [20]	China	3,139 men and women	≥65	CVD mortality (114), all-cause mortality (529)	9.1 years; 27,289 person years	Dietary calcium: M: <458, 458 to 584, 584 to 762, >762; W: <417, 417 to 529, 529 to 688, >688; Calcium supplement users versus nonusers	Age, smoking, sex, BMI, PASE, alcohol, education, history of diabetes and hypertension, energy intake, percentage of energy from total fat, percentage of energy from saturated fat, and calcium supplemental use	7
Dai *et al*. 2013, SWHS [21]	China	73,232 women	40 to 70	CVD mortality (1,147), all-cause mortality (3,806)	11 years; 806,549 person years	Dietary calcium: <408, 408 to 600, ≥600	Age, smoking, sex, BMI, physical activity, alcohol, education, marriage status, tea drinking, use of ginseng, use of calcium supplement, use of multivitamin, intakes of total energy, SFA, phosphorus, fiber, retinol, vitamin E, folic acid, sodium, potassium and zinc	7
Dai *et al*. 2013, SMHS [21]	China	61,414 men	40 to 74	CVD mortality (800), all-cause mortality (2,418)	5.5 years; 336,984 person years	Dietary calcium: <408, 408 to 600, ≥600	Same as above	7
Langsetmo *et al*. 2013, CaMos [28]	Canada	9,033 men and women	≥25	All-cause mortality (1,160)	10 years; 77,558 person years	Dietary calcium: per 500	Age, study center, education, BMI, health status, smoking, alcohol, physical activity, sun exposure, self-reported comorbidity (in men and women: hypertension, heart disease, stroke, type 2 diabetes, COPD, and kidney stones; in women only: osteoporosis, thyroid disease, IBD, breast cancer, and uterine cancer; in men only: prostate cancer), and medication (aspirin use or other NSAIDs)	7
Michaëlsson *et al*. 2013, Swedish mammography cohort [22]	Sweden	61,433 women	39 to 74	CVD mortality (3,862), all-cause mortality (11,944)	19 years; 1,094,880 person years	Dietary calcium: <600, 600 to 999, 1,000 to 1,399, ≥1,400; any calcium users versus nonusers	Age, smoking, BMI, physical activity, total energy and vitamin D intake, a healthy dietary pattern, height, living alone, education, use of calcium-containing supplements, and Charlson’s comorbidity index	8
Xiao *et al*. 2013, NIH-AARP study [23]	USA	388,229 men and women	50 to 71	CVD mortality (11,778)	12 years; 3,549,364 person years	Dietary calcium: M: 478, 616, 739, 898, 1,247 (medians); W: 408, 532, 648, 798, 1,101 (medians); Calcium supplement: 0, <400, 400 to 1,000, ≥1,000	Age, BMI, smoking, race, physical activity, alcohol, education, marital status, health status, supplemental calcium intake, fruit and vegetable intake, red meat intake, whole grain intake, total fat intake, and total caloric intake, and use of menopausal hormone therapy (for women)	8
Van Hemelrijck *et al*. 2013, NHANES III [24]	USA	18,714 men and women	≥17	CVD mortality (1,870)	18 years; 243,227 person years	Dietary calcium: <500, 500 to 1,000, 1,000 to 1,300, >1,300	Age, smoking, BMI, sex, race/ethnicity, physical activity, alcohol, poverty to income ratio, comorbidity index, serum vitamin D, vigorous and total energy intake	7
Li *et al*. 2012, Heidelberg Cohort [25]	German	23,980 men and women	35 to 64	CVD mortality (267)	11 years; 263,780 person years*	Dietary calcium: 513, 675, 820, 1,130 (medians); Calcium only users versus nonusers of supplements	Age, sex, smoking, BMI, physical activity, alcohol, education, history of diabetes, use of calcium supplements, and intakes of total energy, vitamin D, SFAs and total protein	7
Mursu *et al*. 2011, IWHS [27]	USA	38,772 women	5 to 69	CVD mortality (3,319)	11 years; 228,085 person years	Calcium supplement use (yes/no)	Age, education, place of residence, diabetes mellitus, high blood pressure, BMI, waist to hip ratio, hormone replacement therapy, physical activity, smoking, and intake of energy, alcohol, saturated fatty acids, whole grain products, and fruits, and vegetables	7
Kaluza *et al*. 2010, COSM [5]	Sweden	23,366 men	4 to 79	CVD mortality (819), all-cause mortality (2,358)	10 years; 224,206 person years	Dietary calcium: <1230, 1230 to 1598, ≥1599	Age, smoking, physical activity, alcohol, marital status, education, health status, waist-to-hip ratio, energy-adjusted dietary fiber, SFA, vitamin D, and phosphorus intake	7
Umesawa *et al*. 2006, JACC [18]	Japan	21,068 men and 32,319 women	4 to 79	CVD mortality (800)	9.6 years; 515,029 person years	Dietary calcium: M: 250, 363, 449, 536, 665 (medians); W: 266, 379, 462, 545, 667 (medians)	Age, smoking, BMI, alcohol, hypertension, diabetes, and intakes of total energy	6
Van der Vijver *et al*. 1992, Civil Servants [26]	Netherlands	1,340 men and 1,265 women	4 to 65	CVD mortality (NA), all-cause mortality (NA)	28 years; 72,940 person years*	Dietary calcium: M: ≤585, 585 to 1245, >1245; W: ≤445, 445 to 850, >850	Age, energy intake, systolic blood pressure	5

^a^Person time estimated by multiplying number of participants by average follow-up time. BMI, body mass index; CaMos, Canadian Multicentre Osteoporosis Study; COPD, chronic obstructive pulmonary disease; COSM, Cohort of Swedish Men; CVD, cardiovascular disease; IBD, inflammatory bowel disease; IWHS, Iowa Women’s Health Study; JACC, Japan Collaborative Cohort study; M, men; NA, not available; NHANES III, Third National Health and Nutrition Examination Survey; NIH-AARP, National Institutes of Health - AARP Diet and Health Study; NSAIDs, aspirin use or other non-steroidal anti-inflammatory drugs; PASE, Physical Activity Scale for the Elderly; SFA, saturated fatty acid; SMHS, Shanghai Men’s Health Study; SWHS, Shanghai Women’s Health Study; W, women.

One study used one week food frequency recall to measure dietary calcium intake, and all other studies used food frequency questionnaires. All studies provided the estimates adjusted for age. Most of the studies also controlled for energy intake and smoking (n = 10), alcohol consumption and body mass index (n = 9), physical activity (n = 8), and other dietary variables or nutrients (n = 7). No study scored the highest level of quality (maximum 9), with five of the nine studies scoring 8, five scoring 7, and one scoring 6.

### Dietary calcium intake and cardiovascular mortality

Cardiovascular mortality was evaluated in nine prospective cohort studies [5,18,20-26] comprising 709,499 subjects and at least 21,457 deaths from CVD. The exact number of deaths is unknown because one study [26] did not report the number of deaths.

**Figure 2:**
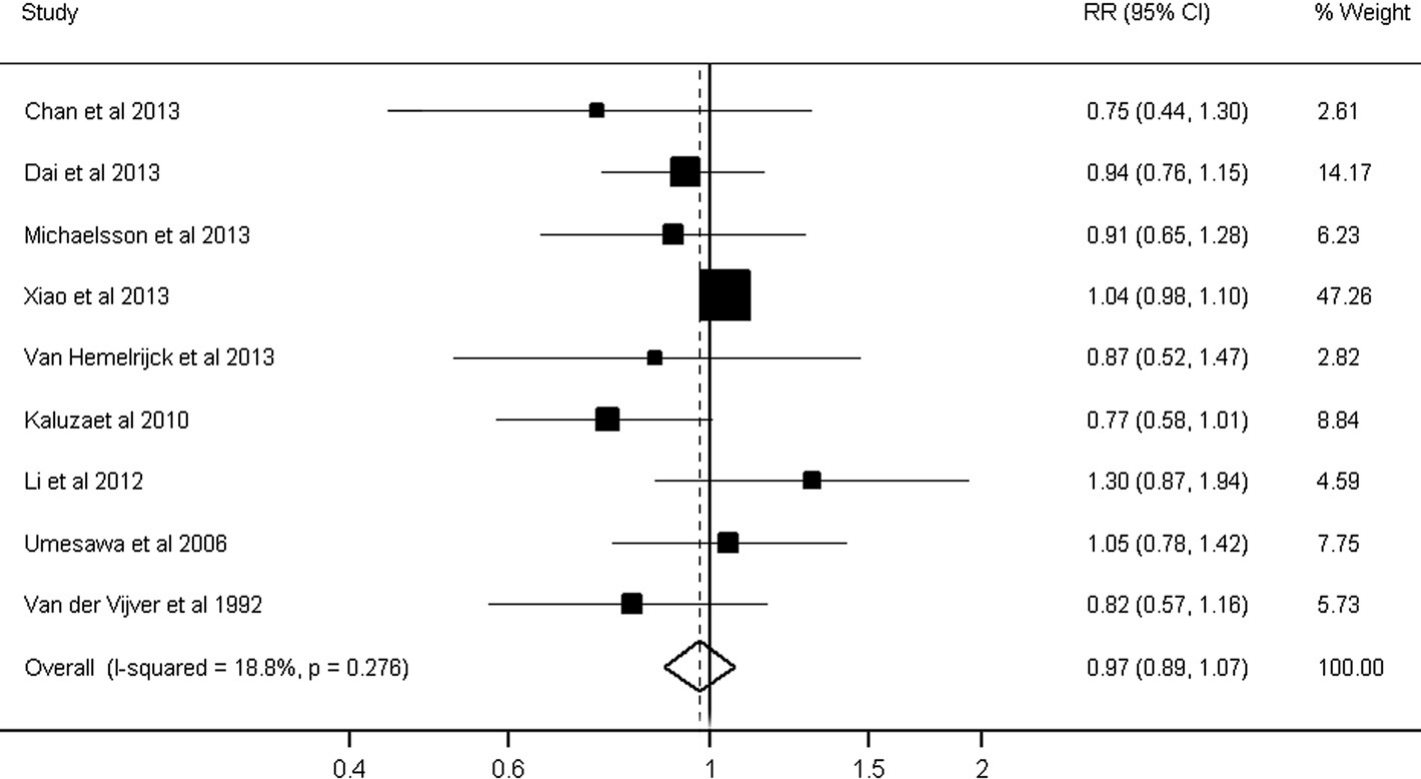
**Dietary calcium intake and risk of cardiovascular mortality.** Forest plot presents association between dietary calcium intake and risk of cardiovascular mortality when comparing the highest to the lowest level of dietary calcium intake. CI, confidence interval; RR, relative risk.

The pooled RR of cardiovascular mortality comparing the highest and lowest level of dietary calcium intake was 0.97 (95% CI: 0.89 to 1.07; *P* = 0.60), with no heterogeneity among the studies (*I*^*2*^ = 18.8%; *P* = 0.28) (Figure 2). The Begg rank correlation test and Egger linear regression test indicated no significant publication bias (Begg, *P* = 0.75; Egger, *P* = 0.11).

**Figure 3:**
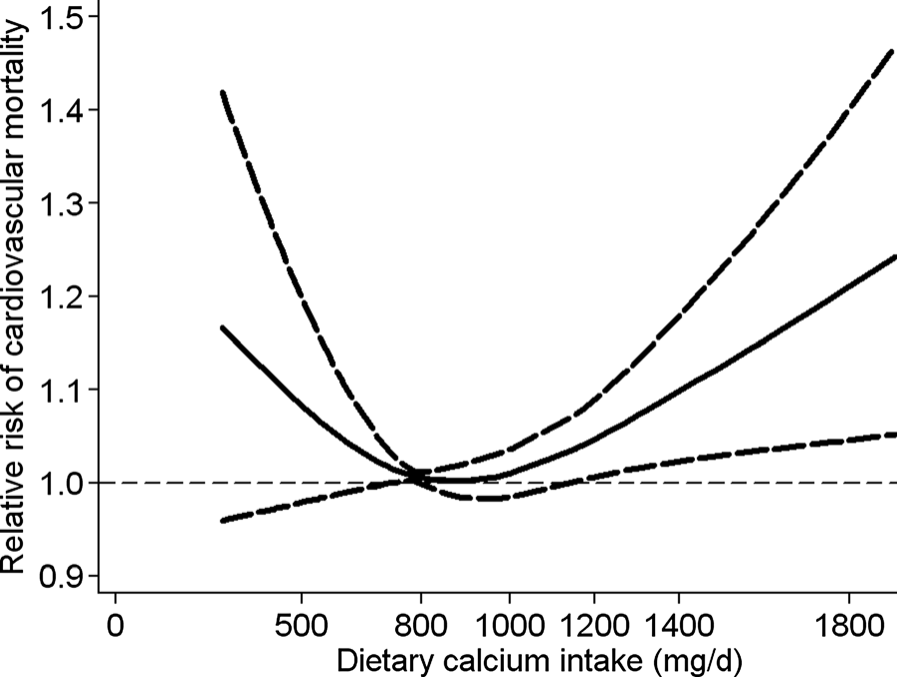
**Dose-response analyses relating dietary calcium intake to cardiovascular mortality.** Relative risks of cardiovascular mortality associated with total dietary calcium intake. Calcium intake was modeled with restricted cubic splines by a random-effects dose-response model. A calcium intake of 800 mg/d was used as the reference to estimate all relative risks.

The dose-response analysis revealeda non-linear association (*P* <0.01). The tendency of a U-shaped association between dietary calcium intake and deaths from CVD was visualized by the pattern of the spline curves in Figure 3. At intakes below 800 mg/day, there was a higher risk of cardiovascular mortality with lower intake of dietary calcium. Intakes around 800 mg/day conferred the lowest risk of cardiovascular mortality. By contrast, at intakes above 800 mg/day, a higher intake was progressively associated with a higher risk of cardiovascular mortality. Compared to individuals with 800 mg*/*day of dietary calcium intake, the predicted RRs for cardiovascular mortality were 1.08 (95% CI: 0.98 to 1.20) for individuals with 500 mg*/*day of calcium intake, 1.01 (95% CI: 0.98 to 1.04) for 1,000 mg/day, 1.05 (95% CI: 1.01 to 1.09) for 1,200 mg/day, and 1.10 (95% CI: 1.02 to 1.18) for 1,400 mg/day.

### Dietary calcium intake and all-cause mortality

All-cause mortality was investigated in six studies [5,20-22,26,28] involving a total of 225,189 subjects and at least 21,055 deaths from all causes. One study [28] was not included in our analysis because it reported only one intake level of calcium. The summary relative risks of all-cause mortality comparing the highest and lowest level of dietary calcium intake was 0.83 (95% CI: 0.70 to 1.00; *P* = 0.05), with significant heterogeneity among the studies (*I*^*2*^ = 74.9%; *P* = 0.003; Additional file 2: Figure S1). No significant publication bias was observed (Begg test, *P* = 0.46; Egger test, *P* = 0.34). We found evidence of a non-linear association (*P* <0.01; Additional file 2: Figure S2). We found a threshold effect at intakes of about 900 mg/day. Compared to intakes of 900 mg/day, a lower intake was gradually associated with a higher risk of all-cause mortality. At intakes above 900 mg/day, risk of all-cause mortality did not reduced with the increase of dietary calcium intake.

### Supplemental calcium intake and cardiovascular mortality

Six studies [20,22-25,27] reported the relationship between supplemental calcium intake and cardiovascular mortality (Table 1). None of the studies found a statistically significant association. Use of calcium supplements was not significantly associated with cardiovascular mortality in comparison with non-use of any supplements (RR = 0.96; 95% CI: 0.82 to 1.13; *P* = 0.66; Additional file 2: Figure S3).

### Subgroup and sensitivity analyses

To test the robustness of the results and investigate the sources of between-study heterogeneity, we conducted subgroup analyses. Table 2 presents the different subgroup analyses of studies on cardiovascular and all-cause mortality. The associations between dietary calcium intake and risk of cardiovascular and all-cause mortality did not differ substantially by study location, sex, or whether vitamin D status was controlled for in models.

**Table 2:** **Stratified analysis on the associations of dietary calcium intake and mortality from cardiovascular disease and all causes**

	**Cardiovascular mortality**					**All-cause mortality**		
	**Number**	**RR (95% CI)**	***P*** ^**a**^	***I*** ^***2***^ **(%)**	**Number**	**RR (95% CI)**	***P*** ^**a**^	***I*** ^***2***^ **(%)**
**Location:**								
United States	2	1.04 (0.98 to 1.10)	0.50	0.0	-	-	-	-
Europe	4	0.90 (0.73 to 1.12)	0.20	36.1	3	0.85 (0.68 to 1.06)	0.08	61.1
Asia	3	0.95 (0.81 to 1.12)	0.56	0.0	2	0.80 (0.52 to 1.23)	0.002	89.9
**Sex:**								
Male and female	3	0.99 (0.70 to 1.39)	0.22	33.3	2	0.66 (0.53 to 0.82)	0.45	0.0
Male	5	0.88 (0.74 to 1.06)	0.06	55.2	2	0.91 (0.74 to 1.11)	0.14	54.7
Female	5	1.04 (0.95 to 1.13)	0.90	0.0	2	0.90 (0.63 to 1.29)	0.001	90.6
**Follow-up time:**								
>10 years	6	1.03 (0.97 to 1.08)	0.46	0.0	3	0.98 (0.89 to 1.07)	0.56	0.0
≤10 years	3	0.87 (0.70 to 1.08)	0.28	21.5	2	0.71 (0.60 to 0.83)	0.26	22.0
**Controlling for vitamin D status in models:**								
Yes	4	0.92 (0.74 to 1.15)	0.22	32.7	2	0.87 (0.65 to 1.16)	0.03	80.0
No	5	1.02 (0.97 to 1.08)	0.44	0.0	3	0.79 (0.57 to 1.10)	0.006	80.7

^a^ *P* for heterogeneity. CI, confidence interval; RR, relative risk.

For all-cause mortality, subgroup analyses showed that dietary calcium intakes were not significantly associated with an increased mortality risk in studies with more than 10 years of follow-up, but not in studies with less than 10 years.

We also performed a sensitivity analysis. Exclusion of one study [20] that enrolled people aged over 65 years yielded similar results for cardiovascular mortality (RR = 1.00; 95% CI: 0.94 to 1.07; *P* = 0.90) or all-cause mortality (RR = 0.89; 95% CI: 0.76 to 1.05; *P* = 0.18).

## Discussion

This meta-analysis of prospective studies indicates a U-shaped relationship between dietary calcium intake and cardiovascular mortality. Compared with intakes of 800 mg/day, both lower and higher intakes were gradually associated with a higher risk of cardiovascular mortality. For all-cause mortality, we also observed a threshold effect at intakes about 900 mg/day. Intakes above 900 mg/day were not associated with a decrease in risk of all-cause mortality.

Vitamin D, directly or indirectly, enhances renal conservation of the absorbed calcium and intestinal absorption of calcium [29]. Some studies have noted that serum 25-hydroxyvitamin D levels, the major circulating metabolite of vitamin D, are inversely correlated with CVD incidence rates [30,31]. The co-administration of calcium with vitamin D may reduce the adverse effect of dietary calcium. Therefore, to explore a potential source of heterogeneity in our results, we also considered vitamin D status associated with dietary calcium intake. However, subgroup analyses showed that dietary calcium intakes were not significantly associated with all-cause mortality in studies that adjusted for vitamin D status.

Heterogeneity could also be caused by differences in dietary magnesium intake. None of the included studies adjusted for dietary magnesium intake. However, some studies showed that dietary magnesium intake is associated with reduced mortality from CVD [32,33]. It is possible that magnesium counters the effects of calcium on risk of CVD. Furthermore, some studies also suggest that balance between calcium and magnesium may help prevent CVD [21,34].

Another possible explanation for the differences between the studies might be the effects of sex and age on the risks of CVD and mortality rates with respect to dietary calcium or supplemental calcium intake. Supplemental calcium intake is associated with an elevated CVD mortality in men but not in women [23]. A recent study by Paik *et al*. [35] found that supplement calcium intake did not increase CVD risk in women. Dietary supplement use is more common in women than in men [36]. Additionally, male users of calcium supplements may start taking calcium supplements at an older age [23]. However, in the present study, sensitivity analyses by excluding one study in older people showed similar results.

Women with a calcium intake below 600 mg/day had a higher risk of stroke in the Nurses’ Health Study [37]. There was higher mortality from ischemic heart disease with a total calcium intake below 700 mg/day in the Iowa Women’s Health Study cohort [4]. An intake below about 500 mg/day was also associated with higher rate of stroke in Japanese people with habitual low calcium intake [18]. In our study, at intakes below 800 mg/day, there was a higher risk of cardiovascular mortality with lower intake of calcium. Our results also indicated that intakes of about 800 mg/day, which corresponds to the recommended daily intake for Swedish women aged more than 50 years [38], conferred the lowest risk of cardiovascular mortality. Moreover, a recent meta-analysis by Larsson *et al*. [39] showed that high dietary calcium intake is related to a lower risk of stroke in populations with low to moderate calcium intakes. Therefore, increased dietary calcium intake may be associated with reduced cardiovascular mortality risk at low to moderate calcium intakes.

In addition, our study showed that, in comparison with intakes of 800 mg/day, a higher intake was associated with increased risk of cardiovascular mortality. In a re-analysis of randomized trials, a higher rate of myocardial infarction was observed for calcium supplementation [40]. The results from a meta-analysis of randomized trials of calcium supplements also indicated a higher risk of cardiovascular events in women with intake levels of dietary calcium above 800 mg/day, but not in women with lower intake levels [6]. Thus, it is likely that increasing dietary calcium intake may increase risk of cardiovascular mortality in individuals who already consume adequate amounts of calcium.

Our results are concordant with findings from a recent review [41] in which calcium intake was not associated with risk of CVD when comparing the highest and lowest intake levels. However, this study [41] did not evaluate the dose-response relationship of dietary calcium intake and CVD. In the current study, dose-response analysis showed that the association between dietary calcium intake and cardiovascular mortality was U-shaped. The null findings to date for comparing the highest and lowest calcium intake categories likely related to the non-linear association.

Moreover, a recent meta-analysis [42] showed that higher calcium intake may be associated with a reduction in risk of colorectal cancer beyond 1,000 mg/day. There could therefore be beneficial effects of calcium intake on risk of some cancers, such as colorectal. However, a meta-analysis of randomized controlled trials suggested no effect of calcium on the risk of total cancer [43]. In the present study, a high calcium intake (>900 mg/day) was not associated with a decreased rate of all-cause mortality. We found that, for all-cause mortality, dietary calcium intakes were not significantly associated with an increased mortality risk in studies with more than 10 years of follow-up, but not in studies with less than 10 years. It is possible that the follow-up periods in these studies were too short to identify the true associations with mortality. In fact, longer induction periods were observed for cancers than for CVD.

For calcium supplement use, a previous review [44] suggested that the evidence regarding the relationship between calcium supplement use and increased CVD risk is insufficient. A study by Radford *et al*. [45] showed that the effects of calcium supplements on cardiovascular risk did not differ across varying patient subpopulations, such as younger people and those with low dietary calcium intake. The present study also found no evidence that calcium supplements increased the risk of cardiovascular mortality. Thus, more longitudinal studies are needed to confirm or refute the results of our study.

Calcium is a vital electrolyte and part of the etiological pathway of CVD. Nevertheless, calcium has mainly been explored in relation to bone health. As a concern in osteoporosis management, it is only recently that the cardiovascular safety of calcium supplementation has been queried [46]. Calcium blood concentrations are under tight homeostatic control by the calciotropic hormones [47]. Alterations in calcium homeostasis caused by diets that are low or very high in calcium can alter blood levels of calcium and calciotropic hormones [48]. Calcium intakes that are too low may cause mortality or CVD risk through pathways that affect blood pressure [49,50], insulin secretion and sensitivity [51-53], and blood cholesterol concentrations [53]. Likewise, excessive amounts of calcium may also exert a harmful effect on cardiovascular health by inducing a hypercoagulable state [54,55]. Many studies show that high levels of circulating fibroblast growth factor-23 are associated with higher risk of cardiovascular events and all-cause mortality [56-58], whereas calcium-enriched meals can raise serum levels of fibroblast growth factor-23 [59]. High calcium intake may also increase risk of mortality associated with vascular and soft tissue calcification [60,61] and effects on arterial stiffness [62,63].

We used estimates from the fully adjusted models from all included studies in our analyses to reduce the potential for confounding. We conducted the dose-response meta-analysis to evaluate non-linear relationships, which helped to test the shape of these possible associations. Some limitations of this meta-analysis should be addressed. All included studies were observational in nature. The results may be subject to residual confounding or unmeasured factors. Moreover, dietary calcium intake was measured at baseline in the included studies. During the long follow-up, participants may have changed their diets, including changing their dietary calcium intakes. Therefore, we were not able to evaluate change in dietary calcium intake during follow-up. Besides, calcium intake levels were assessed by food frequency questionnaires in most studies. Measurement errors of calcium intake were possible. However, the prospective nature of the included studies could have led to an underestimation of the real association and could not explain the positive associations we observed in this study.

Our results, together with previous studies, suggest that increasing dietary calcium intake is associated with decreased mortality risk at low to moderate calcium intakes, whereas it is not associated with a decreased risk of mortality at high calcium intakes. Thus, intake recommendations for calcium should consider individual characteristics and should focus on people with low intake levels of calcium, rather than increasing the intake of those with adequate amounts of calcium.

## Conclusions

This meta-analysis suggests a U-shaped relationship between dietary calcium intake and risk of cardiovascular mortality. A high calcium intake (>900 mg/day) was not associated with a reduced risk of all-cause mortality.

## Additional files

Additional file 1:
**Meta-analysis of Observational Studies in Epidemiology Checklist.**
Additional file 2: Figure S1. Association between dietary calcium intake and risk of all-cause mortality when comparing the highest to lowest level of dietary calcium intake. **Figure S2.** Dose-response analyses relating dietary calcium intake to all-cause mortality. RRs of all-cause mortality associated with dietary calcium intake. Calcium intake was modeled with restricted cubic splines by a random-effects dose-response model. A calcium intake of 500 mg/d was used as the reference to estimate all RRs. **Figure S3.** Association between calcium supplement use and risk of cardiovascular mortality when comparing users of calcium supplements with non-users.

## Abbreviations

CI: confidence interval
CVD: cardiovascular disease
RR: relative risk
