# Supplementary material for: Dietary calcium intake and mortality risk from cardiovascular disease and all causes: a meta-analysis of prospective cohort studies
Source: BMC Med. 2014 Sep 25;12:158. doi: 10.1186/s12916-014-0158-6 (PMC4199062; doi:10.1186/s12916-014-0158-6)
Supplement: Additional file 1: — Meta-analysis of Observational Studies in Epidemiology Checklist. [file 12916_2014_158_MOESM1_ESM.doc]

**MOOSE Checklist**

**Dietary** **Calcium Intake and** **Risk of Mortality from Cardiovascular Disease and All Causes: A Meta-Analysis of Prospective Cohort Studies**

Xia Wang, MD

School of Public Health, Shandong University

Hongxia Chen, MD

Institute of Biomedicine, Taihe Hospital, Hubei University of Medicine, Shiyan, Hubei Province, China

Yingying Ouyang PhD and Jun Liu PhD

Department of Nutrition and Food Hygiene, School of Public Health, Tongji Medical College, Huazhong University of Science and Technology

Gang Zhao, MD

Shandong Provincial Hospital affiliated to Shandong University

Corresponding Author:

Wei Bao, MD, PhD

Department of Nutrition and Food Hygiene, School of Public Health, Tongji Medical College, Huazhong University of Science and Technology, 13 Hangkong Road, Wuhan 430030, China, tel:301 435 2295 fax: 301 435 2295; ([wei.bao@nih.gov](mailto:wei.bao@nih.gov)).

| **Criteria** | | **Brief description of how the criteria were handled in the meta-analysis** |
| --- | --- | --- |
| **Reporting of background should include** | |  |
| √ | Problem definition | Considerable controversy exists regarding the association between dietary calcium intake and risk of mortality from cardiovascular disease and all causes. |
| √ | Hypothesis statement | Increasing dietary calcium intake was associated with reduced mortality risk at low to moderate calcium intake, while it may increase mortality risk at high calcium intake. |
| √ | Description of study outcomes | Total and cardiovascular mortality. |
| √ | Type of exposure or intervention used | Dietary calcium intake. |
| √ | Type of study designs used | We only included prospective cohort studies. |
| √ | Study population | We placed no restriction. |
| **Reporting of search strategy should include** | |  |
| √ | Qualifications of searchers | The credentials of the three investigators YyO, JL, and GZ are indicated in the author list. |
| √ | Search strategy, including time period included in the synthesis and keywords | Medline (1950 to present), Embase (1980 to present), Web of knowledge (1970 to present)  See figure 1. |
| √ | Databases and registries searched | Pubmed, Embase, and Web of knowledge |
| √ | Search software used, name and version, including special features | We did not employ a search software. EndNote was used to merge retrieved citations and eliminate duplications. |
| √ | Use of hand searching | We hand-searched bibliographies of retrieved papers for additional references. |
| √ | List of citations located and those excluded, including justifications | Details of the literature search process are outlined in the flow chart. The citation list is available upon request. |
| √ | Method of addressing articles published in languages other than English | We placed no restrictions on language. |
| √ | Method of handling abstracts and unpublished studies | We did not found unpublished studies on the association. |
| √ | Description of any contact with authors | We contacted the authors by email to obtain additional data for the meta-analysis. |
| **Reporting of methods should include** | |  |
| √ | Description of relevance or appropriateness of studies assembled for assessing the hypothesis to be tested | Detailed inclusion and exclusion criteria were described in the methods section. |
| √ | Rationale for the selection and coding of data | Data extracted from each of the studies were relevant to the population characteristics, study design, exposure, outcome, and possible effect modifiers of the association. |
| √ | Assessment of confounding | Conducted sensitivity analyses. |
| √ | Assessment of study quality, including blinding of quality assessors; stratification or regression on possible predictors of study results | Study quality was evaluated by using the Newcastle-Ottawa quality assessment scale. The system allowed a total score of 0-9 points (9 representing the highest quality) |
| √ | Assessment of heterogeneity | Heterogeneity of the studies were explored within two types of study designs using Cochrane’s Q test of heterogeneity and I^2^ statistic that provides the relative amount of variance of the summary effect due to the between-study heterogeneity. |
| √ | Description of statistical methods in sufficient detail to be replicated | Description of methods of meta-analyses, sensitivity analyses and assessment of publication bias are detailed in the methods. |
| √ | Provision of appropriate tables and graphics | We included detailing the terms used for database search, 1 flow chart, 2 summary table, 5 forest plot of all studies. |
| **Reporting of results should include** | |  |
| √ | Graph summarizing individual study estimates and overall estimate | Figures 2 and 3, and additional file 2: Figure S1-S3 |
| √ | Table giving descriptive information for each study included | Table 1 |
| √ | Results of sensitivity testing | In text |
| √ | Indication of statistical uncertainty of findings | 95% confidence intervals were presented with all summary estimates, I^2^ values and results of sensitivity analyses |
| **Reporting of discussion should include** | |  |
| √ | Quantitative assessment of bias | Subgroup analyses and sensitivity analyses indicate heterogeneity in strengths of the association due to most common biases in observational studies. |
| √ | Justification for exclusion | We excluded the studies if they had a case-control or cross-sectional design. |
| √ | Assessment of quality of included studies | We discussed the results of the analyses. |
| **Reporting of conclusions should include** | |  |
| √ | Consideration of alternative explanations for observed results | We discussed that potential unmeasured confounders may have caused residual confounding. We noted that the variations in the strengths of association may be due to true population differences, or to differences in quality of studies. |
| √ | Generalization of the conclusions | Findings from this meta-analysis indicate that a U-shape relationship between dietary calcium intake and risk of cardiovascular mortality. |
| √ | Guidelines for future research | We recommend future studies to confirm the effect of dietary calcium intake. |
| √ | Disclosure of funding source | Supported by grants from National Natural Science Foundation (NSFC 81370966) of China. The sponsors played no role in the design of the study; in the collection, analysis, or interpretation of the data; or in the preparation, or approval of the manuscript. |
