# Supplementary figures and images for: Dietary calcium intake and mortality risk from cardiovascular disease and all causes: a meta-analysis of prospective cohort studies
Source: BMC Med. 2014 Sep 25;12:158. doi: 10.1186/s12916-014-0158-6 (PMC4199062; doi:10.1186/s12916-014-0158-6)

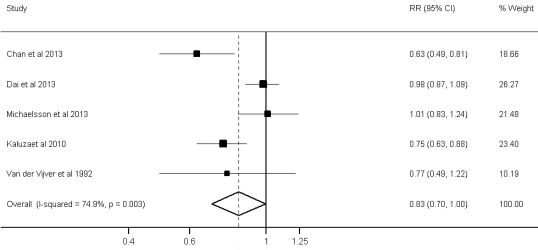

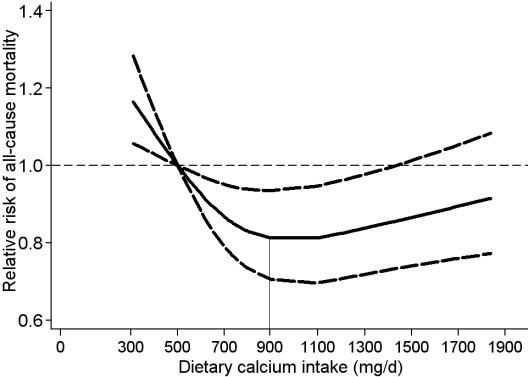

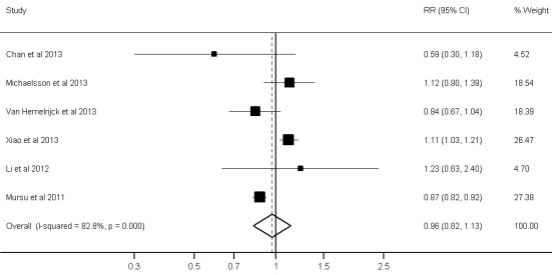

Supplement: Additional file 2: Figure S1. — Association between dietary calcium intake and risk of all-cause mortality when comparing the highest to lowest level of dietary calcium intake. Figure S2. Dose-response analyses relating dietary calcium intake to all-cause mortality. RRs of all-cause mortality associated with dietary calcium intake. Calcium intake was modeled with restricted cubic splines by a random-effects dose-response model. A calcium intake of 500 mg/d was used as the reference to estimate all RRs. Figure S3. Association between calcium supplement use and risk of cardiovascular mortality when comparing users of calcium supplements with non-users. [file 12916_2014_158_MOESM2_ESM.pdf]
